# A Conservation Roadmap for the Anticipated Eurasian lynx (*Lynx lynx*) Metapopulation in Central Europe—Modelling Effective Deme Sizes and Connectivity

**DOI:** 10.1111/eva.70340

**Published:** 2026-10-24

**Authors:** Gregor Rolshausen, Christine Breitenmoser, Jarmila Krojerová‐Prokešová, Tomaž Skrbinšek, Carsten Nowak

**Affiliations:** ^1^ Conservation Genetics Group & Centre for Wildlife Genetics Senckenberg Nature Research, Research Station Gelnhausen Gelnhausen Germany; ^2^ Foundation KORA Ittigen Switzerland; ^3^ Czech Academy of Sciences Institute of Vertebrate Biology Brno Czech Republic; ^4^ Department of Zoology, Fisheries and Hydrobiology, Faculty of AgriSciences Mendel University in Brno Brno Czech Republic; ^5^ Biotechnical Faculty University of Ljubljana Ljubljana Slovenia; ^6^ DivjaLabs Ltd. Ljubljana Slovenia; ^7^ LOEWE Centre for Translational Biodiversity Genomics (LOEWE‐TBG) Frankfurt am Main Germany

## Abstract

Reintroducing wildlife into anthropogenic landscapes is challenging, particularly for wide‐ranging species. Extensive habitat fragmentation and limited carrying capacities pose genuine risks of population isolation and subsequent genetic erosion. A current example includes the fragmented populations of the Eurasian lynx (*Lynx lynx carpathicus*) in Central Europe, where isolated demes currently rely on ex situ sourced or wild‐caught releases to buffer inbreeding accumulation over short timescales. Due to management trade‐offs, however, such assisted migration can only function as a transitional remedy, whereas long‐term stability hinges on inherent growth rates and vital genetic exchange in a metapopulation framework. We here use analytic modelling to examine potential scenarios and requirements for a vital metapopulation of the Eurasian lynx in Central Europe. To inform conservation management in the early stages of formation, we considered scenarios that describe the current and near‐future status of the metapopulation, as well as anticipated—but not yet established—scenarios that would ensure long‐term genetic stability. We find that current and near‐future effective sizes and migration rates among demes are far from sufficient to reach long‐term stability (Ne_meta_ > 500), even given hundreds of generations. Model‐derived management targets suggest that robust configurations require significant increases in deme sizes (> 150%), diversity (Ne/Nc > 0.4), and average pairwise migration rates (> 10%), in order to advance towards stable metapopulation dynamics. This, however, entails potential distribution trade‐offs from limited capacities in *ex situ* and/or wild population sourcing. Here, our model outcomes caution against diverting resources towards new introductions, and highlight the need for ongoing reinforcement of existing demes on the one hand, and the mitigation of dispersal barriers and threats on the other hand.

## 1 Introduction

Species reintroduction as a central tool in biodiversity conservation and ecological restoration aims to reestablish viable populations of formerly lost species in their natural environments (Seddon et al. 2007; Armstrong and Seddon 2008). However, habitat space that is suitable for reintroduction is often embedded in a matrix of anthropogenic land use, characterised by high patchiness and low connectivity (Fletcher et al. 2024). As a consequence, impaired genetic exchange will lead to mosaic population structures that further promote local inbreeding and subsequent genetic impoverishment (Delaney et al. 2010; Haddad et al. 2015; Lewis et al. 2015). Similar threats associated with low connectivity intensify in reintroduced populations that are bound to grow from wild‐caught or captive‐bred individuals, already starting with low effective population sizes (Hoban et al. 2013; Seddon et al. 2014; Taylor et al. 2017). Conservation management of reintroduced populations thus follows a network approach that involves two central goals: (i) to establish viable local subpopulations (demes) in suitable habitat patches, and (ii) to connect demes among each other via gene flow to balance local inbreeding effects and foster a viable metapopulation (Armstrong 2005; Hodgson et al. 2009; Gilbert and Whitlock 2015; Ryman et al. 2019). Accordingly, these goals require both ongoing evaluation of local population health via genetic monitoring, as well as extrapolation of (meta)population health based on anticipated increases in local genetic diversity and connectivity among demes.

A metapopulation is a network of demes that occupy disjunct habitat patches and are connected via migration (Hastings and Harrison 1994; Hanski and Simberloff 1997; Hanski and Ovaskainen 2003). Akin to a single isolated population, metapopulations can be characterised as units of conservation that are susceptible to the loss of genetic diversity and the accumulation of inbreeding. In fact, as demes connect in a network and exchange migrants, their (local) genetic diversity now hinges on both internal dynamics, such as inbreeding and drift, as well as external dynamics, such as gene flow and the genetic diversity of connected demes (Waples 2010; Gilbert and Whitlock 2015; Ryman et al. 2019). Correspondingly, a genetically effective population size—depicting the size of an ideal Wright‐Fisher population with a distinct diversity and drift dynamic (Ne, Wright 1931) – can also be specified for a metapopulation with a distinct diversity and drift dynamic (Ne_meta_, Laikre et al. 2016, Ryman et al. 2019). More precisely, Ne_meta_ describes the rate of loss of heterozygosity in a given metapopulation, denoting it as the size of an ideal substructured population sustaining the same loss rate, while taking into account each deme's (local) Ne together with all pairwise migration rates between demes (Hössjer et al. 2014, 2015). Practical to conservation genetic monitoring, Ne_meta_ can then be interpreted according to the established “50/500 guideline” (Franklin 1980; Jamieson and Allendorf 2012): As a short‐term benchmark, Ne_meta_ > 50 is necessary to avoid critical inbreeding; and as a long‐term benchmark, Ne_meta_ > 500 is necessary to retain genetic variation in quantitative traits associated with fitness (Jamieson and Allendorf 2012; Hössjer et al. 2016; Laikre et al. 2016; Ryman et al. 2019). Particularly in reintroduced or reconnected population networks that are typically vulnerable to local inbreeding and rely on external gene flow, Ne_meta_ can serve as a projection tool to evaluate and refine ongoing management. We here use Ne_meta_ evaluation to investigate the recolonisation status and future prospect of the Eurasian lynx (*Lynx lynx carpathicus*) in Central Europe.

The Eurasian lynx is distributed widely across the eastern Eurasian continent but has suffered severe decline and population extinctions in Central and Western Europe. Remaining autochthonous populations are still found in Scandinavia, the Baltic and the Carpathian region, along with wide‐ranging populations in Russia and Asia (Bazzicalupo et al. 2022; Lucena‐Perez et al. 2020). In addition, ongoing reintroduction efforts dating back to the 1970s have successfully established several managed populations in Central Europe, sourced from the wild Carpathian population, as well as captive‐breeding programs (Bonn Lynx Expert Group 2021). However, despite evidence for demographic growth, genetic diversity is low and inbreeding is critical in most of the reintroduced populations (Breitenmoser‐Würsten and Obexer‐Ruff 2003; Gajdárová et al. 2023; Mueller et al. 2022; Sindičić et al. 2013). Moreover, although suitable habitat is available for reintroductions (Schadt et al. 2002; Kramer‐Schadt et al. 2004; Iannella et al. 2024; Oeser et al. 2023), the long‐term persistence of genetically viable lynx populations in Central Europe's mountain ranges will continue to rely on reinforcement and vital genetic exchange in a metapopulation framework (Bonn Lynx Expert Group 2021).

The current strategic framework for the restoration of a viable lynx metapopulation in Central Europe (Figure 1) comprises the large autochthonous Carpathian population (Ca) alongside six reintroduced subpopulations in the upper Rhine Valley (UR), the western Alps (wA), the eastern Alps (eA), the Dinaric region (Di), the Bohemian Forest region (BBA), and the Harz Mountains (Hz). In addition, recent reintroduction programmes in Germany have released lynx in the Black Forest region (BF), Thuringian Forest (TH), and the Ore Mountains (OM). Release locations were chosen based on evaluation of suitable habitat criteria such as patch size, forest cover, and potential connectivity (Schadt et al. 2002; Oeser et al. 2023). However, effective migration between subpopulations is still low and the connectivity to the Carpathian source population is not established (Bonn Lynx Expert Group 2021). Moreover, several subpopulations, including the most recent releases, have not reached demographic stability and still rely on assisted migration to counter local inbreeding.

**FIGURE 1:**
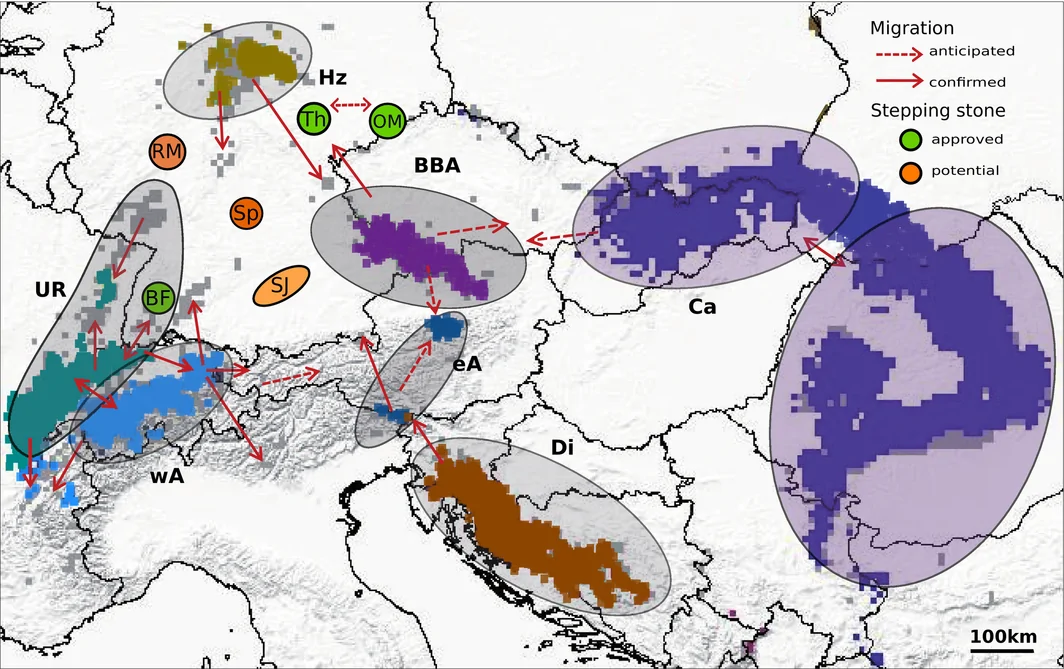
Distribution of Eurasian lynx demes in the anticipated metapopulation network in Central Europe. Established demes, including the Carpathian core population (Ca), are highlighted with grey ellipses. Recent stepping stone introductions are depicted in green, and potential future introductions are depicted in orange. Arrows indicate anticipated and confirmed migration events. BBA, Bohemian Forest region; BF, Black Forest; Di, Dinaric region; eA, Eastern Alps; Hz, Harz mountains; OM, Ore Mountains; RM, Rhön Mountains; SJ, Swabian Jura; SP, Spessart Forest; Th, Thuringian Forest; UR, Upper Rhine Valley; wA, Western Alps. For a detailed Europe‐wide distribution map see Kaczensky et al. (2024).

At this early stage of the anticipated metapopulation, it is important to better understand the basic requirements regarding genetic diversity and migration rates among all connected demes. In this work, we address this research objective by examining model‐based predictions of effective metapopulation size (Ne_meta_) and the accumulation of inbreeding over time (Δ*f*) for particular conservation scenarios (Table 1). Given the spatial distribution of suitable habitat patches and their anticipated connectivity (Figure 1), we address objectives regarding requirements in migration, demographic stability, and additional management interventions.

**TABLE 1:** Overview over the ten scenarios that were considered in our models. Scenarios differ in the number of demes connected to the metapopulation, and sub‐scenarios (1–4) differ in migration rates and deme sizes (see respective Figure for size values). Migration rates depict values for the low “l”, medium “m”, and high “h” category (“na” indicates absence of that category in the model). Nc_meta_ refers to an estimate of the accumulated census size of all introduced demes, and its proportional increases in respective scenarios. Note that Sc2.1–2.4 modify primarily the three stepping‐stone demes, whereas Sc3.1–3.4 involve increases in Nc across the metapopulation (excluding the autochthonous Carpathian population). Modelled effective metapopulation size is reported after 50 generations (Ne_meta50_), and at mutation‐drift equilibrium (Ne_metaE_).

		Demes	Stepping stones	Migration rates	Nc_meta_	Ne_meta50_	Ne_metaE_	
Sc1		UR, wA, eA, BBA, Hz, Di	—	0.01, 0.05, 0.1	600–700	92.4	453.8	Figure 2
Sc2	1	UR, wA, eA, BBA, Hz, Di	BF, Th, OM	0.01, 0.05, 0.1	+15%	110.6	482.9	Figure 2
	2	UR, wA, eA, BBA, Hz, Di	BF, Th, OM	0.015, 0.075, 0.15	+50%	154.5	495.8	Figure S1A
	3	UR, wA, eA, BBA, Hz, Di	BF, Th, OM	0.02, 0.1, 0.2	+40%	142.1	390.4	Figure S1B
	4	UR, wA, eA, BBA, Hz, Di	BF, Th, OM	0.03, 0.15, 0.3	+30%	143.7	318.2	Figure S1C
Sc3	1	UR, wA, eA, BBA, Hz, Di, Ca	BF, Th, OM	na, 0.05, 0.1	+90%	344.9	538.9	Figure 3
	2	UR, wA, eA, BBA, Hz, Di, Ca	BF, Th, OM	na, 0.1, 0.2	+150%	565.1	668.7	Figure 3
	3	UR, wA, eA, BBA, Hz, Di, Ca	BF, Th, OM	0.02, 0.1, 0.2	+40%	196.4	390.7	Figure S2A
	4	UR, wA, eA, BBA, Hz, Di, Ca	BF, Th, OM	na, 0.1, 0.2	+90%	416.2	534.1	Figure S2B
Sc4		UR, wA, eA, BBA, Hz, Di, Ca	BF, Th, OM, SP, SJ, RM	0.01, 0.05, 0.1	+120%	401.9	616.7	Figure 4

Abbreviations: BBA, Bohemian Forest region; BF, Black Forest; Di, Dinaric region; eA, Eastern Alps; Hz, Harz mountains; OM, Ore Mountains; RM, Rhön Mountains; SJ, Swabian Jura; SP, Spessart Forest; Th, Thuringian Forest; UR, Upper Rhine Valley; wA, Western Alps.

## 2 Methods

### 2.1 Modelling Ne_meta_ and Δ*f*

The concept of a genetically effective population size has been mathematically extended from a single isolated population to complex subdivided populations by Hössjer et al. (2014, 2015). We here use an implementation of this analytical framework (gesp, Olsson et al. 2017) that allows for exact computation of inbreeding accumulation (Δ*f*) and effective population size (Ne_meta_) over time in metapopulations with varying local deme effective sizes (Ne_x_), census sizes (Nc), and migration rates (m) among demes. Our models consider diploid non‐selfing populations with mating after migration and migration rates (m) denoted as proportions of the population that migrates. Following the example of Pacín et al. (2024) we established four effective migration rates as a baseline for evaluation: low (0.01), medium (0.05), high (0.1), and no migration (0)—describing the proportion of individuals in the respective deme that migrate. Migration rates were then assigned to all pairs of demes based on geographical distance and patch connectivity (Schadt et al. 2002; Kramer‐Schadt et al. 2004; Bonn Lynx Expert Group 2021; Iannella et al. 2024; Sánchez‐Arribas et al. 2026). Ne_meta_ is expressed here as inbreeding effective size, ignoring mutation and selection while assuming deme sizes to be constant. The rate at which identical‐by‐descent (IBD) probabilities of randomly drawn gene pairs increases (i.e., inbreeding accumulation) is therefore determined by migration and drift only (Hössjer et al. 2015; Olsson et al. 2017). Initial kinship coefficients and inbreeding accumulated in demes during their founding phases were ignored. Ne_meta_ was further weighted by local effective sizes of demes, such that each deme's contribution to Ne_meta_ is proportional to its size. We also computed the eigenvalue effective size (Ne_metaE_, Hössjer et al. 2014), depicting the effective size of the metapopulation reached at drift‐migration equilibrium, which requires time‐invariant deme sizes and that no deme is isolated. For a particular scenario, Ne_metaE_ can then be interpreted as the long‐term limit effective size of the metapopulation.

Results for each scenario are then shown as (i) the progression of effective sizes for each deme (Ne_x_) and the metapopulation (Ne_meta_) over time to examine whether Ne_metaE_ reaches the anticipated long‐term benchmark of values > 500 (Jamieson and Allendorf 2012), and the number of generations required to do so; and (ii) the accumulation of inbreeding (Δ*f*) in demes and in the metapopulation over a period of 20 generations to evaluate the short‐term benchmark of Ne_meta_ > 50, corresponding to an effective population size where inbreeding does not increase by more than 1% per generation.

### 2.2 Scenarios

We investigated four main scenarios with relevance to short‐ and long‐term conservation of the lynx metapopulation in Central Europe. The focus of the investigation was to explore important milestones for the establishment of the metapopulation: Scenario 1 (Sc1) describes the anticipated state of the core metapopulation currently under consideration. Geneflow among demes is expressed in a pairwise migration matrix based on a connected habitat mosaic, and deme sizes reflect local near‐future census targets. Initially, the underlying habitat mosaic does not include stepping stones and lacks connectivity to the autochthonous Carpathian population (Ca). Migration rates are asymmetric and chosen to reflect criteria such as patch size, forest cover, and potential connectivity (Schadt et al. 2002; Oeser et al. 2023). Scenario 2 (Sc2) expands the mosaic, describing the anticipated core state now further connected to three recently approved stepping stone reintroductions in the Black Forest region (BF), Thuringian Forest (Th), and Ore Mountains (OM). The comparison between Sc1 and Sc2 allows an evaluation of the impact of the three stepping stones on the metapopulation, as well as initial demographic requirements for these stepping stones. Scenario 3 (Sc3) addresses plausible future conditions, describing the connected metapopulation in Central Europe with an established link to the northern margins of the autochthonous Carpathian population (Ca). Notably, the connection to the Carpathian population is anticipated to establish in the most northern part of the Ca distribution in the Czech Republic and Slovak Republic, and only via the BBA deme (Figure 1). We therefore assumed the initial deme size for Ca in the models to be lower (Nc = 400) than the currently reported overall size of the Carpathian population as a whole (Nc > 2500, https://www.lcie.org/large‐carnivores/eurasian‐lynx). Scenario 4 (Sc4) addresses the hypothetical extension of Sc3 where three potential future introductions in Germany (SP: Spessart Forest, SJ: Swabian Jura, RM: Rhön Mountains) are connected to the metapopulation in Central Europe. Sc4 was considered to explore basic demographic requirements for future stepping stone introductions.

Scenarios were chosen to reflect a varying number of demes within the range of local carrying capacities and patch connectivity. Deme sizes were estimated based on empirical estimates from included populations (Fležar et al. 2023; Gajdárová et al. 2023; Krojerová‐Prokešová et al. 2023; Pazhenkova et al. 2025; Serva et al. 2025; Skrbinšek et al. 2019; Wölfl et al. 2021), as well as expert consultation (www.kora.ch, Bonn Lynx Expert Group 2021). Deme effective sizes were further chosen based on a Ne/Nc ratio of ~0.4 (mean = 0.44, SD = 0.08) to fall within the range of reported values for other felids (Diefenbach et al. 2015; Janecka et al. 2008; Pacín et al. 2024; Smith and McDougal 1991). We constructed ten models (Table 1) intended to explore a range of conceivable combinations of these factors with the aim to yield efficient recommendations that would allow a genetically robust metapopulation. Moreover, because Ne/Nc ratios strongly depend on local drift dynamics (Hoban et al. 2024; Mastretta‐Yanes et al. 2024), small and isolated populations of Eurasian lynx might fall below commonly reported Ne/Nc ratios. We therefore also included models of Sc1–Sc3 that were based on a rather conservative ratio (Ne/Nc = 0.15), in order to evaluate the robustness of our findings against the background of low initial genetic diversity in demes.

## 3 Results

The evaluation of different conservation scenarios for the Central European lynx metapopulation revealed that none of the currently developing scenarios will comply with the proposed long‐term conservation targets in the near future. Namely, for the contemporary scenarios Sc1 and Sc2—both still pending full implementation—even a long period of > 800 generations did not suffice to reach Ne_meta_ > 500, regardless of the additional reintroductions (BF, Th, and OM) being connected to the core metapopulation or not (Figure 2). In fact, the Sc1 and Sc2 configurations were predicted to also fall below Ne_meta_ > 500 at drift‐migration equilibrium (Ne_metaE,Sc1_ = 453.8, Ne_metaE,Sc2.1_ = 482.9). Moreover, for a more realistic long‐term timeline of approximately 50 generations, Ne_meta_ barely exceeded 100 (Ne_meta50,Sc1_ = 92.4, Ne_meta50,Sc2.1_ = 110.6, Figure 2). However, the accumulation of inbreeding (Δ*f*_meta_) over 20 generations for Sc1 and Sc2 progressed within the margins of the short‐term genetic benchmark of Ne_meta_ > 50 (i.e., Δ*f*_meta_ ≤ 0.01), with demes Hz and Di, as well as the stepping stones (BF, Th, and OM) failing to reach that reference target. Alternative parameterisation for the Sc2 models, assuming higher overall migration rates and/or larger deme sizes for the stepping stones (Sc2.2–2.4, Table 1), also fell short of significantly improving the situation. In particular, a threefold increase in deme size for the stepping stones, together with a 1.5× increase in migration rates, still failed to meet the long‐term requirements of Ne_meta_ > 500 (Ne_meta50,Sc2.2_ = 154.5, with Ne_metaE,Sc2.2_ = 495.8), yet succeeded to meet the short‐term requirements of Ne_meta_ > 50 over 20 generations (Figure S1). Further increases in migration rates (Sc2.3: m × 2, Sc2.4: m × 3) did lead to a faster convergence of Ne_meta_ to its expected value at drift‐migration equilibrium (less than 500 generations to reach Ne_metaE_) but also reduced that value significantly (all Ne_metaE_ < 400, Figure S1, Table 1).

**FIGURE 2:**
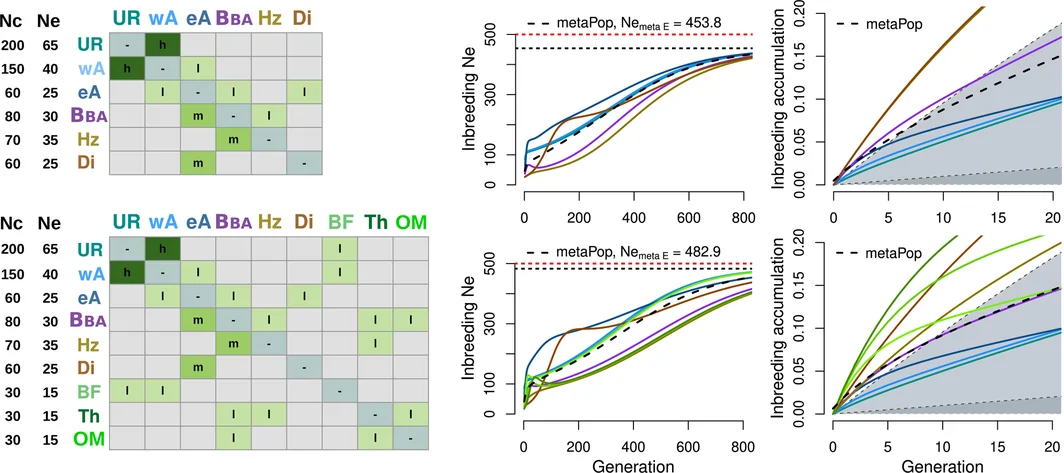
Model outcomes for Sc1 (upper panel) and Sc2 (lower panel). First column: Pairwise migration matrix and initial parameters (Nc, Ne), acronyms correspond to main text. Migration rates indicated as low “l”, medium “m”, and high “h” correspond to Table 1 (*x*/*y* depicting from/to direction). Second column: Lines depict effective size (Ne_x_) predictions over time, for demes (solid), and the metapopulation (dashed). Horizontal lines depict Ne_500_ (red dots), and the equilibrium effective size Ne_metaE_ of the respective scenario (black dots). Third column: Lines depict inbreeding accumulation over 20 generations in demes (solid), and the metapopulation (dashed); envelopes mark the trajectory of a population of Ne_50_ (light grey) and of Ne_500_ (dark grey) indicating the short‐ and long‐term conservation goal. Colors and acronyms correspond to Figure 1.

Explorative modelling of Sc3—which connects the Carpathian deme (Ca) to the core metapopulation—was then carried out to find parsimonious parameter sets that would comply with the proposed conservation targets. Here, we found that an overall increase in migration dynamics upgrading low‐rates (*m* = 0.01) to medium‐rates (*m* = 0.05), and medium‐rates (*m* = 0.05) to high‐rates (*m* = 0.1), together with a 90% increase in Nc among demes (excluding the Ca deme), was necessary to reach Ne_meta_ > 500 in approximately 200 generations with a drift‐migration equilibrium size of Ne_metaE,Sc3.1_ = 538.9 and Ne_meta50,Sc3.1_ = 344.9 after 50 generations (Figure 3 and Figure S2). To reach Ne_meta_ > 500 already within 50 generations, another doubling of migration rates was necessary, together with a 150% increase in the overall Nc_meta_ (Ne_metaE,Sc3.2_ = 668.7 and Ne_meta50,Sc3.2_ = 565.1, Figure 3). Similarly to Sc2.3 and Sc2.4, an increase in migration rates that was not accompanied by substantial increases in Nc_meta_ (> 90%) did not suffice to reach Ne_meta_ > 500 for the metapopulation (Ne_metaE,Sc3.3_ = 390.7, Ne_metaE,Sc3.4_ = 534.1, Table 1). The short‐term benchmark of Δ*f*_meta_ ≤ 0.01 was achieved with all parameter sets for Sc3 (Figure 3 and Figure S2). Additional models for Sc1 and Sc2 that were based on a more conservative Ne/Nc ratio (Ne/Nc = 0.15) showed a significant impact on the short‐term benchmark of Ne > 50 in all demes, with only the UR and wA demes reaching Δ*f*_meta_ ≤ 0.01 after 20 generations; as well as on the long‐term benchmark of Ne_metaE_ > 500, which was not achieved for any of the scenarios (Figure S3). Sc3 models based on the conservative Ne/Nc ratio further showed significant decreases in equilibrium sizes (all Ne_metaE_ < 400, Figure S4).

**FIGURE 3:**
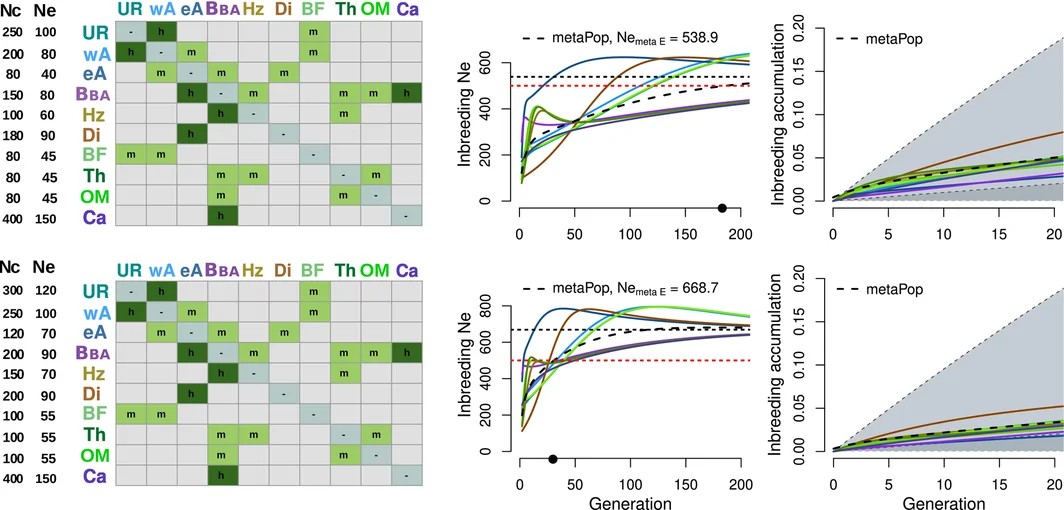
Model outcomes for Sc3 with moderate increase in gene flow (upper panel), and strong increase in gene flow (lower panel, see Table 1). First column: Pairwise migration matrix. Second column: Effective size (Ne_x_) predictions over time. The black dot on the *x*‐axis marks the timepoint for the metapopulation to reach the long‐term benchmark of Ne_500_. Third column: Inbreeding accumulation over time. Details, see caption Figure 2.

Finally, exploring the incorporation of three additional stepping stones (Sp, SJ, and RM) into the metapopulation (Sc4) revealed that rather large (Nc > 60) and connected (*m* > 0.01) stepping stone populations are needed to contribute positively to the overall development of the metapopulation (Ne_metaE,Sc4_ = 616.7 and Ne_meta50,Sc4_ = 401.9, Figure 4).

**FIGURE 4:**
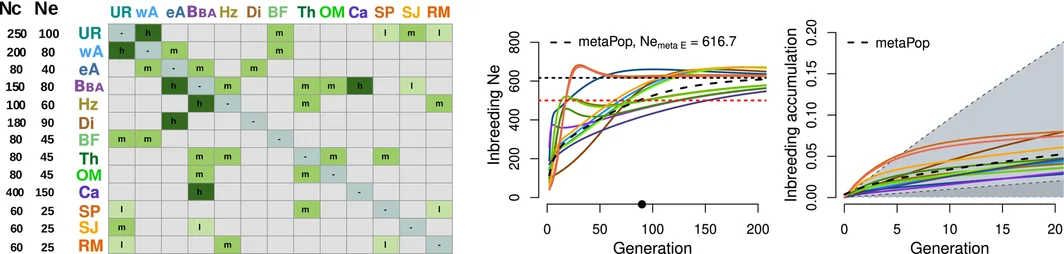
Model outcomes for Sc4, an ideal scenario that includes three additional stepping stones. First column: Pairwise migration matrix. Second column: Effective size (Ne_x_) predictions over time. The black dot on the *x*‐axis marks the timepoint for the metapopulation to reach the long‐term benchmark of Ne_500_. Third column: Inbreeding accumulation over time. Details, see caption Figure 2.

## 4 Discussion

The envisioned lynx metapopulation in Central Europe is currently still in a formative state. Gene flow between the six core demes is just starting to establish with very few dispersers between the Harz Mountains (Hz) and the Bohemian Forest region (BBA), as well as occasional dispersers between the upper Rhine Valley (UR) and the western Alps (wA). The eastern Alps (eA) and the Dinaric region remain largely isolated (Krofel et al. 2024). The near‐future goal is thus to establish migration between demes, particularly enhancing the connection between the Dinaric region (Di), the eastern Alps (eA), and the western Alps (wA) on the one hand, and the connection between the Harz Mountains (Hz) and the Bohemian Forest region (BBA) on the other hand. The latter goal is further supported by the recent introduction of two additional stepping‐stones in Thuringian Forest (Th) and Ore Mountains (OM). In addition to improved genetic exchange among the isolated reintroduced populations, a central long‐term goal is the connection of the Western Carpathian population (Ca) with the Central European metapopulation via the pivotal BBA deme (Figure 1). Implementing these goals requires ongoing distribution of captive‐bred or wild‐caught individuals into demes, and/or translocations between demes, in order to advance local effective sizes and connectivity (Bonn Lynx Expert Group 2021). As a first guideline, we here provide a model‐based assessment of conceivable deme sizes and natural migration rates that were predicted sufficient to maintain metapopulation genetic diversity and ensure its long‐term viability.

We found that the current deme sizes and near‐future projected migration rates (Sc1) are far too low to reach the long‐term conservation benchmark of Ne_meta_ > 500 within a reasonable timeframe of ~50 generations (e.g., Ne_meta50,Sc1_ = 92.4, Ne_meta50,Sc2.1_ = 110.6). In addition, isolated populations, such as those in the Harz Mountains (Hz) and the Dinaric region (Di), as well as the recently established stepping stones (BF, Th, and OM), are threatened by high rates of short‐term inbreeding accumulation over the next 20 generations (Sc2, Figure 2). Here, mitigation would require migration rates to exceed 0.05 or at least two effective migrants per generation in the stepping stones, and more than three effective migrants for the established Di and Hz demes. Accordingly, in order to approach a robust long‐term genetic viability perspective for all demes, both deme census sizes and migration rates have to be substantially increased (Table 1, Figure S1). More specifically, Sc3 predicted beneficial migration rates between the eastern Alps (eA) and the Dinaric region (Di) ranging between three and nine migrants per generation, approximately eight migrants per generation between the upper Rhine Valley (UR) and the western Alps (wA), and more than four migrants per generation between the Harz Mountains and the Bohemian Forest region (BBA). The projected connection of the Carpathian population (Ca) via the BBA deme in the Bohemian Forest region (Sc3) required more than ten migrants per generation in order to contribute beneficially to the overall diversity of the metapopulation. A robust long‐term conservation benchmark of Ne_meta_ > 500 within 50–100 generations (Figure 3 and Figure S2) would further involve vital connectivity between the pivotal BBA deme and the eastern Alps deme (eA), as well as with the stepping stones Th and OM, and the Hz deme. In general, the stepping stone demes (BF, Th, and OM) would need to be connected to their respective sinks and sources by approximately two to three migrants per generation. Moreover, the demes from the Dinaric region and the eastern Alpine region have to, at least, double in effective size (from 20 < Ne_x_ < 35 to 70 < Ne_x_ < 90), and the stepping stones have to be sourced up to threefold of their currently anticipated effective sizes in order to maintain their diversity under local drift dynamics (i.e., from Ne_x_ < 20 to Ne_x_ > 45). Eventually, the connection between the western Alps (wA) and the eastern Alps (eA) has to be established with more than three migrants per generation. However, the feasibility of bridging the large dispersal distance from the western to the eastern Alps will likely depend on the establishment of additional stepping stones in the future.

The magnitude of Ne_metaE_ has been shown to generally depend on effective deme sizes rather than migration rates, whereas the time to reach Ne_metaE_ in a metapopulation mainly depends on migration rates, with higher rates allowing faster accomplishment. Inbreeding accumulation, however, depends on both deme effective size and migration (Pacín et al. 2024). Ongoing reinforcement efforts thus have to account for a balance between (i) provisionally reinforcing isolated demes to thwart local inbreeding and extinction, and (ii) sufficiently supporting stepping stone demes to establish migration dynamics. Particularly in metapopulations that strongly deviate from their drift‐migration equilibrium, isolated demes under threat of extinction can cause complex sink dynamics due to asymmetric migration that in turn threaten both the remaining demes, as well as the metapopulation as a whole (Hedrick and Gilpin 1997; Waples 2010; Kurland et al. 2023; Olusanya et al. 2025). These dynamics are further pronounced under linear stepping‐stone connectivity where not all demes directly exchange migrants and gene flow is restricted to specific corridors (Kurland et al. 2023; Thomas et al. 2025)—as is the case for the Central European lynx metapopulation. Given the prevailing threat of extinction for particular populations in Central Europe—such as the eastern Alpine (eA) and the Dinaric (Di) demes (Sindičić et al. 2013; Fležar et al. 2021; Pazhenkova et al. 2025), the Jura Mountains (Bull et al. 2016; Vogt et al. 2025), or the Vosges‐Palatinian region (Vandel et al. 2006; Gimenez et al. 2019; Port et al. 2024) – management efforts should aim at reinforcing these demes to catalyze genetic rescue and mitigate the risk of sink dynamics at the metapopulation level (Kurland et al. 2023; Pazhenkova et al. 2025; Bauduin et al. 2025).

At the same time, two recently introduced stepping stones, the Thuringian Forest deme (Th) and the Ore Mountains deme (OM), are of distinct importance for the metapopulation. On the one hand, they aim to facilitate the establishment of dispersal‐driven population nuclei north of the core Bohemian Forest region, where sporadic dispersal out of the BBA deme has already been recorded (Gajdárová et al. 2023). Such population outposts are crucial for successful population expansions because they increase the general encounter probability of dispersers. Particularly in subadult lynx, dispersal activity is rather conservative and largely guided by the concurrence of conspecifics, as opposed to competition‐driven density effects in their natal range (Ferreras et al. 2004; Zimmermann et al. 2005; Zimmermann et al. 2007; Iannella et al. 2024). Increasing densities in the Th and OM stepping stones are therefore vital to successful population expansions, both for the Bohemian Forest (BBA) deme, as well as for the Harz Mountains (Hz) deme. On the other hand, the Th and OM demes are anticipated to serve as future migration hubs to attract and further distribute dispersers from the Harz Mountains region as well as from the Bohemian Forest region (Figure 1), thus establishing long‐term gene flow in the eastern part of the metapopulation. Notably, although such population outposts could also establish by themselves via natural dispersal, the imbalance between female and male dispersal makes them particularly vulnerable to demographic stochasticity (Port et al. 2024; Von Arx et al. 2021). At this early stage of formation, ongoing assisted migration is thus needed to eventually increase local effective sizes of the stepping stones to at least Ne_x_ > 20 (or Nc > 50) respectively, in order to thwart local extinction and/or sink dynamics impacting the metapopulation. Moreover, reinforcement should be accompanied by further improvement of landscape permeability and habitat quality in regions flanking the core demes to facilitate dispersal and allow expansion (Zimmermann et al. 2005; Zimmermann et al. 2007; Krojerová‐Prokešová et al. 2019; Iannella et al. 2024). Importantly, the above discussed model estimates and recommendations (i.e., model scenario Sc3, Table 1) critically hinge on similar efforts to improve expansion success and connectivity for the eastern edge of the BBA deme, where the link to the Carpathian (Ca) population is expected (Gajdárová et al. 2021); as well as for the southern edge, where the link to the eastern Alpine (eA) deme should be established, which in turn links the Dinaric (Di) deme to the metapopulation (Favilli et al. 2023; Iannella et al. 2024). Notably, habitat quality and carrying capacities per se are favourable outside of the Bohemian Forest (Müller et al. 2014; Magg et al. 2016), whilst major factors limiting lynx distribution and dispersal are of anthropogenic origin, such as agricultural land use, settlements, roads, and illegal killings (Filla et al. 2017; Heurich et al. 2018; Červený et al. 2019; Ripari et al. 2022; Premier et al. 2025).

The pivotal role of the BBA deme in establishing functional connectivity in the eastern part of the metapopulation (Heurich et al. 2018) is in turn mirrored by the western Alpine (wA) deme in the western part of the metapopulation. In particular, in the northwest of the wA deme, connectivity has to build towards the upper Rhine region (UR), the Black Forest (BF), as well as towards the Jura mountains in the French part of the Alps. The ultimate objective for the wA deme is then to connect to the metapopulation network via the eA deme in the eastern Alps (Bonn Lynx Expert Group 2021). However, despite ongoing efforts to link the wA deme with its neighbouring demes, dispersal and effective migration are still critically low in the Alpine region (Molinari‐Jobin et al. 2010; Gimenez et al. 2019; Iannella et al. 2024).

## 5 Conservation Implications

Taken together, the framework that would adequately support a vital metapopulation capable of buffering the short‐term accumulation of inbreeding (Sc2, Figure S1), and eventually reaching long‐term stability (Sc3, Figure 3), requires careful management to form in practice. As natural gene flow among demes is either not established yet or insufficient to enhance local effective sizes, assisted migration into demes is essential. In particular, it is important to counteract local inbreeding and its potential cascading effects on life history parameters (Auclair et al. 2025) and population health (Geret et al. 2011; Ruiz‐López et al. 2012; Niehaus et al. 2025; Borel et al. 2026). Moreover, in order to avoid complex sink dynamics in the forming metapopulation (Kurland et al. 2023; Olusanya et al. 2025), isolated demes have to become sufficiently vital to advance towards higher connectivity and beneficial gene flow with neighbouring demes. In this regard, Pazhenkova et al. (2025) show promising results for the Dinaric deme, where inbreeding accumulation could be buffered via consecutive releases over a period of 4 years (Krofel et al. 2024). Likewise, our model outcomes suggest that once connectivity is established, inbreeding in small demes can be buffered already in the early stages of the metapopulation, given demes reach sufficiently large effective sizes (Sc2). However, as applied conservation resources are inevitably limited, there will also be trade‐offs among applicable measures. For instance, limited captive breeding capacities may necessitate strategic trade‐offs between augmenting established, yet vulnerable demes and supporting small, recently founded stepping‐stone populations. Importantly, even though assisted migration may successfully trigger genetic rescue in threatened populations (Hogg et al. 2006; Johnson et al. 2010; Godoy et al. 2024; Lopez et al. 2024), the initial positive effect can be rather short‐lived if gene flow is not continuously supplied (Lotsander et al. 2021; Pazhenkova et al. 2025). Moreover, factors such as imbalanced reproductive success or maternal effects among local versus introduced individuals can lead to elevated inbreeding in future generations (Hedrick and Fredrickson 2010, Hedrick et al. 2019, Lotsander et al. 2021). It is thus central to ongoing reinforcement logistics to consider the genetic and demographic viability of the *ex situ* population with regard to a long‐term strategy of continuous assisted migration into threatened demes and stepping stones. In addition to providing sufficient numbers of individuals to avoid pulsed releases that are followed by long periods of isolation, breeding programs need to implement a genetic distribution strategy in order to avoid mating between close relatives and aim at increasing the effective size of the ex situ population. In accordance with genetic monitoring of within‐deme diversity, released lynx should be selected for average kinship minimisation in the respective demes (Caballero and Toro 2000; Kleinman‐Ruiz et al. 2019; Godoy et al. 2025). Moreover, reinforcement should ideally promote philopatry—particularly in small, newly established demes—via resource‐consuming gradual release strategies including an acclimatisation period (i.e., soft release, Maran et al. 2009, Wilson 2018). Yet, the optimisation of release strategies warrants further investigation.

Notably, due to rather optimistic initial assumptions in our models, the implications derived from them likely represent minimal requirements regarding the short‐term (Ne_meta_ > 50) and long‐term (Ne_meta_ > 500) reference targets, rather then precise conservation goals. For instance, the underlying Ne/Nc ratio of ~0.4 might be violated in small demes with large drift effects. Moreover, the models did not address initial kinship coefficients and inbreeding accumulated in demes during their founding phases. Explorative modelling of lower initial starting diversity in demes revealed significant negative effects on short‐term inbreeding accumulation with only the UR and wA demes reaching Δ*f*_meta_ ≤ 0.01 after 20 generations. Also, the long‐term benchmark of Ne_metaE_ > 500 was not achieved for any of the scenarios given a low Ne/Nc ratio (Figures S3 and S4). It is therefore crucial to focus on increasing local deme diversity (i.e., increasing the Ne/Nc ratio) via ongoing reinforcement and kinship minimisation. Moreover, migration rate estimates do not incorporate differences in male and female dispersal behaviour and establishment. Strong male‐biased dispersal in lynx (Samelius et al. 2012; Krojerová‐Prokešová et al. 2019) might therefore require higher effective migration rates than suggested from the models.

The above challenges and trade‐offs imply that diverting management efforts away from existing core demes and stepping stones likely presents a risk to the successful formation of a metapopulation. As introducing new demes, for example, would require substantial local effective sizes (Sc4) to avoid creating metapopulation sinks (Kurland et al. 2023, Olusanya et al. 2025), management should prioritize reinforcement (Sc3) rather than additional introductions in new areas. Notably, population growth in reintroduced populations of the Eurasian lynx has been evidently slow (Vandel et al. 2006; Magg et al. 2016), and successful establishment is burdened by multiple anthropogenic hazards inherent to human‐dominated landscapes (Heurich et al. 2018; Ripari et al. 2022; Premier et al. 2025; Borel et al. 2026). At the same time, the ambitious target of Ne_meta_ > 500 in Central Europe involves robust carrying capacities in the considered habitat patches, as well as significantly improved patch connectivity, both of which are threatened by ongoing fragmentation and land‐use change (Crooks et al. 2017; Fletcher et al. 2024; Premier et al. 2025; Sánchez‐Arribas et al. 2026). Thus, apart from ongoing genetic reinforcement and habitat protection, mitigation efforts are needed to counter anthropogenic threats, such as poaching and traffic mortality outside of protected areas. Indeed, the above discussed target values reflect ideal population growth and dispersal conditions for which implementation is by no means certain. However, considerable variation exists in effective population sizes across taxa with many natural populations currently failing the 50/500 benchmark, particularly in mammals impacted by human activities (Schmidt et al. 2021; Clarke et al. 2024; Mergeay et al. 2024). Currently, it is not well understood under which circumstances populations may well persist naturally at below‐threshold values, and to what degree the spatial scale of population differentiation sets a limit to long‐term conservation goals (Neel et al. 2013, Clarke et al. 2024). Ongoing progress towards stable and expanding lynx populations in Central Europe should therefore not be discouraged by the ambitious target of Ne_meta_ > 500, but rather inform short‐term management efforts based on parameters associated with maximized long‐term stability.

## Funding

The authors have nothing to report.

## Conflicts of Interest

The authors declare no conflicts of interest.

## Supporting information


**Figure S1:** Model outcomes for Sc2, a scenario with the anticipated core state and three connected stepping stones. Panels A–C depict different subscenarios with a 50% increase in census sizes and migration rates relative to Sc1 (Sc2A), a 40% increase in census size and a doubling of migration rates (Sc2B), and a 30% increase in census sizes and a tripling of migration rates (Sc2C). First column: pairwise migration matrix and initial parameters (Nc, Ne), acronyms correspond to main text. Migration rates indicated as low “l”, medium “m”, and high “h” correspond to Table 1 (x/y depicting from/to direction). Second column: Lines depict effective size (Ne_x_) predictions over time, for demes (solid), and the metapopulation (dashed). Horizontal lines depict the Ne_500_ long‐term goal (red dots), and the equilibrium effective size Ne_metaE_ of the scenario (black dots). Third column: Lines depict inbreeding accumulation over 20 generations in demes (solid), and the metapopulation (dashed); envelopes mark the trajectory of a population of Ne_50_ (light grey) and of Ne_500_ (dark grey) indicating the short‐ and long‐term conservation goal.
**Figure S2:** Model outcomes for Sc3, a scenario with the anticipated core state, three connected stepping stones, and the connection to the Carpathian deme (Ca). Panels depict (A) a 40% increase in census sizes and a doubling of migration rates relative to Sc1, and (B) a 90% increase in census sizes and doubling of migration rates and the “low” migration category omitted. First column: pairwise migration matrix and initial parameters (Nc, Ne), acronyms correspond to main text. Migration rates indicated as low “l”, medium “m”, and high “h” correspond to Table 1 (x/y depicting from/to direction). Second column: Lines depict effective size (Ne_x_) predictions over time, for demes (solid), and the metapopulation (dashed). Horizontal lines depict the Ne_500_ long‐term goal (red dots), and the equilibrium effective size Ne_metaE_ of the scenario (black dots). Third column: Lines depict inbreeding accumulation over 20 generations in demes (solid), and the metapopulation (dashed); envelopes mark the trajectory of a population of Ne_50_ (light grey) and of Ne_500_ (dark grey) indicating the short‐ and long‐term conservation goal.
**Figure S3:** Model outcomes for Sc1 (upper panel) and Sc2 (lower panel), based on a Ne/Nc = 0.15 ratio (see main text). First column: pairwise migration matrix and initial parameters (Nc, Ne), acronyms correspond to main text. Migration rates indicated as low “l”, medium “m”, and high “h” correspond to Table 1 (*x*/*y* depicting from/to direction). Second column: Lines depict effective size (Ne_x_) predictions over time, for demes (solid), and the metapopulation (dashed). Horizontal lines depict the Ne_500_ long‐term goal (red dots), and the equilibrium effective size Ne_metaE_ of the respective scenario (black dots). Third column: Lines depict inbreeding accumulation over 20 generations in demes (solid), and the metapopulation (dashed); envelopes mark the trajectory of a population of Ne_50_ (light grey) and of Ne_500_ (dark grey) indicating the short‐ and long‐term conservation goal. Colors and acronyms correspond to Figure 1.
**Figure S4:** Model outcomes for Sc3 with moderate increase in gene flow (upper panel), and strong increase in gene flow (lower panel), based on a Ne/Nc = 0.15 ratio (see main text). First column: pairwise migration matrix with migration rates indicated as low “l”, medium “m”, and high “h” correspond to Table 1 (*x*/*y* depicting from/to direction). Second column: effective size (Ne_x_) predictions over time. Third column: inbreeding accumulation over time. Details, see caption Figure 2.

## Data Availability

The authors have nothing to report.
