# Supplementary material for: A Conservation Roadmap for the Anticipated Eurasian lynx (Lynx lynx) Metapopulation in Central Europe—Modelling Effective Deme Sizes and Connectivity
Source: Evol Appl. 2026 Oct 24;19(10):e70340. doi: 10.1111/eva.70340 (PMC13695031; doi:10.1111/eva.70340)
Supplement: Supplementary file 1 [file EVA-19-e70340-s001.pdf]

A conservation roadmap for the anticipated Eurasian lynx (*Lynx lynx*) metapopulation in Central Europe – modelling effective deme sizes and connectivity.

Supplementary Material

Figure S1

Figure S2

Figure S3

Figure S4

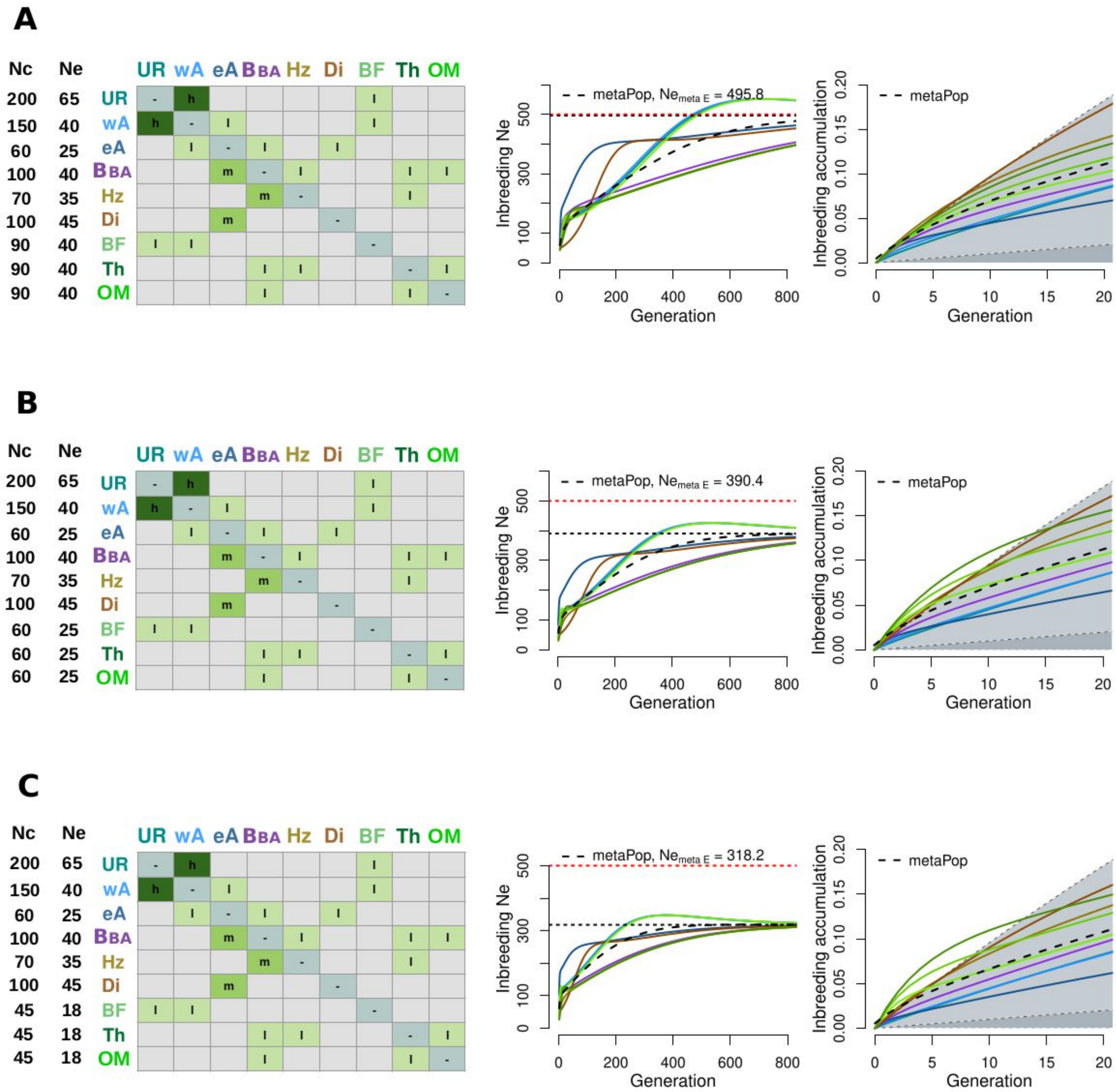

Figure S1 Model outcomes for Sc2, a scenario with the anticipated core state and three connected stepping stones. Panels A-C depict different subscenarios with a 50% increase in census sizes and migration rates relative to Sc1 (Sc2A), a 40% increase in census size and a doubling of migration rates (Sc2B), and a 30% increase in census sizes and a tripling of migration rates (Sc2C). First column: pairwise migration matrix and initial parameters (Nc, Ne), acronyms correspond to main text. Migration rates indicated as low “l”, medium “m”, and high “h” correspond to Table 1 (x/y depicting from/to direction). Second column: Lines depict effective size (Ne_x) predictions over time, for demes (solid), and the metapopulation (dashed). Horizontal lines depict the Ne_{500} long-term goal (red dots), and the equilibrium effective size $Ne_{meta}E$ of the scenario (black dots). Third column: Lines depict inbreeding accumulation over 20 generations in demes (solid), and the metapopulation (dashed); envelopes mark the trajectory of a population of Ne_{50} (light grey) and of Ne_{500} (dark grey) indicating the short- and long-term conservation goal.

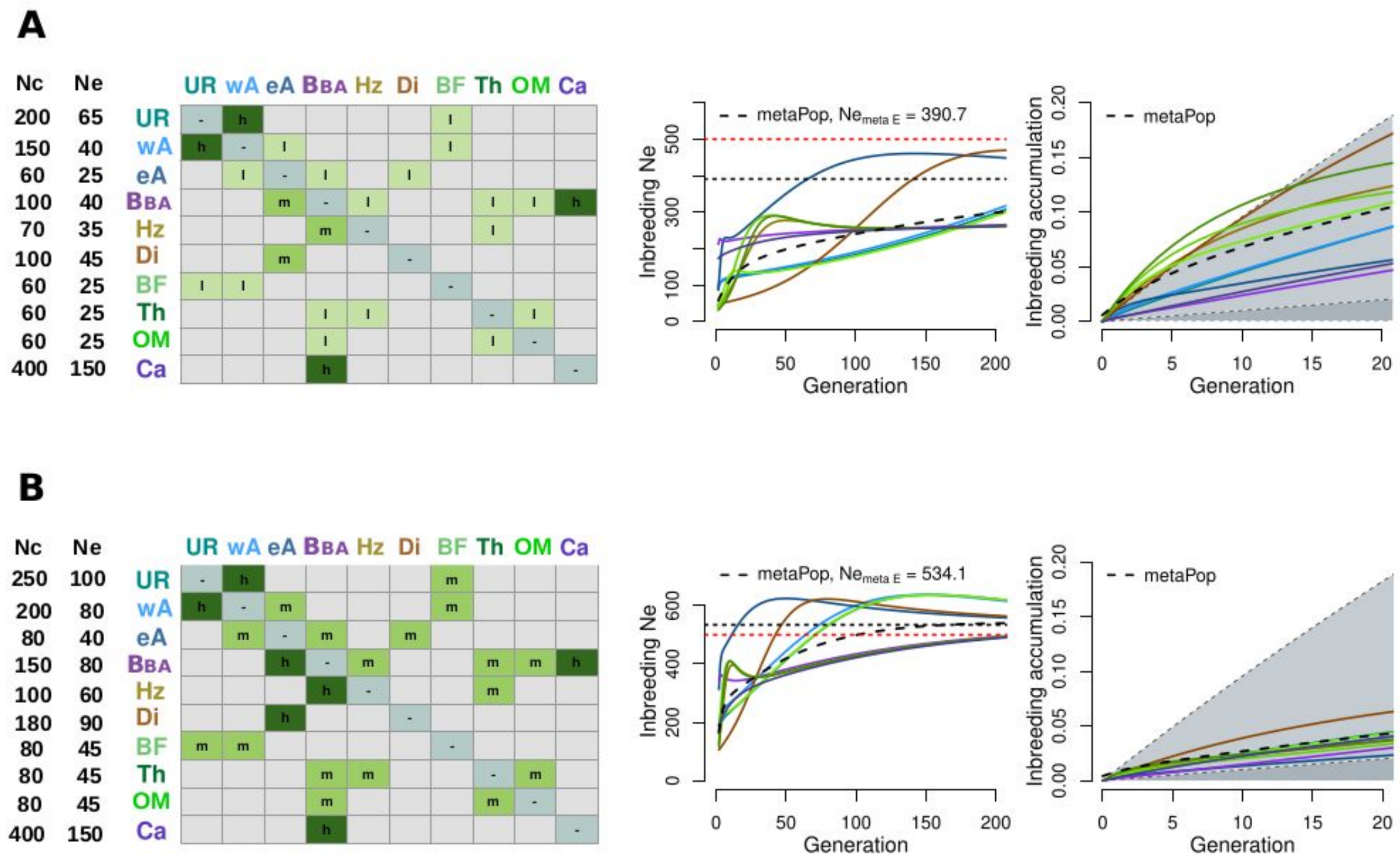

Figure S2 Model outcomes for Sc3, a scenario with the anticipated core state, three connected stepping stones, and the connection to the Carpathian deme (Ca). Panels depict (A) a 40% increase in census sizes and a doubling of migration rates relative to Sc1, and (B) a 90% increase in census sizes and doubling of migration rates and the "low" migration category omitted. First column: pairwise migration matrix and initial parameters (Nc, Ne), acronyms correspond to main text. Migration rates indicated as low "l", medium "m", and high "h" correspond to Table 1 (x/y depicting from/to direction). Second column: Lines depict effective size (Ne_x) predictions over time, for demes (solid), and the metapopulation (dashed). Horizontal lines depict the Ne_{500} long-term goal (red dots), and the equilibrium effective size $Ne_{meta}E$ of the scenario (black dots). Third column: Lines depict inbreeding accumulation over 20 generations in demes (solid), and the metapopulation (dashed); envelopes mark the trajectory of a population of Ne_{50} (light grey) and of Ne_{500} (dark grey) indicating the short- and long-term conservation goal.

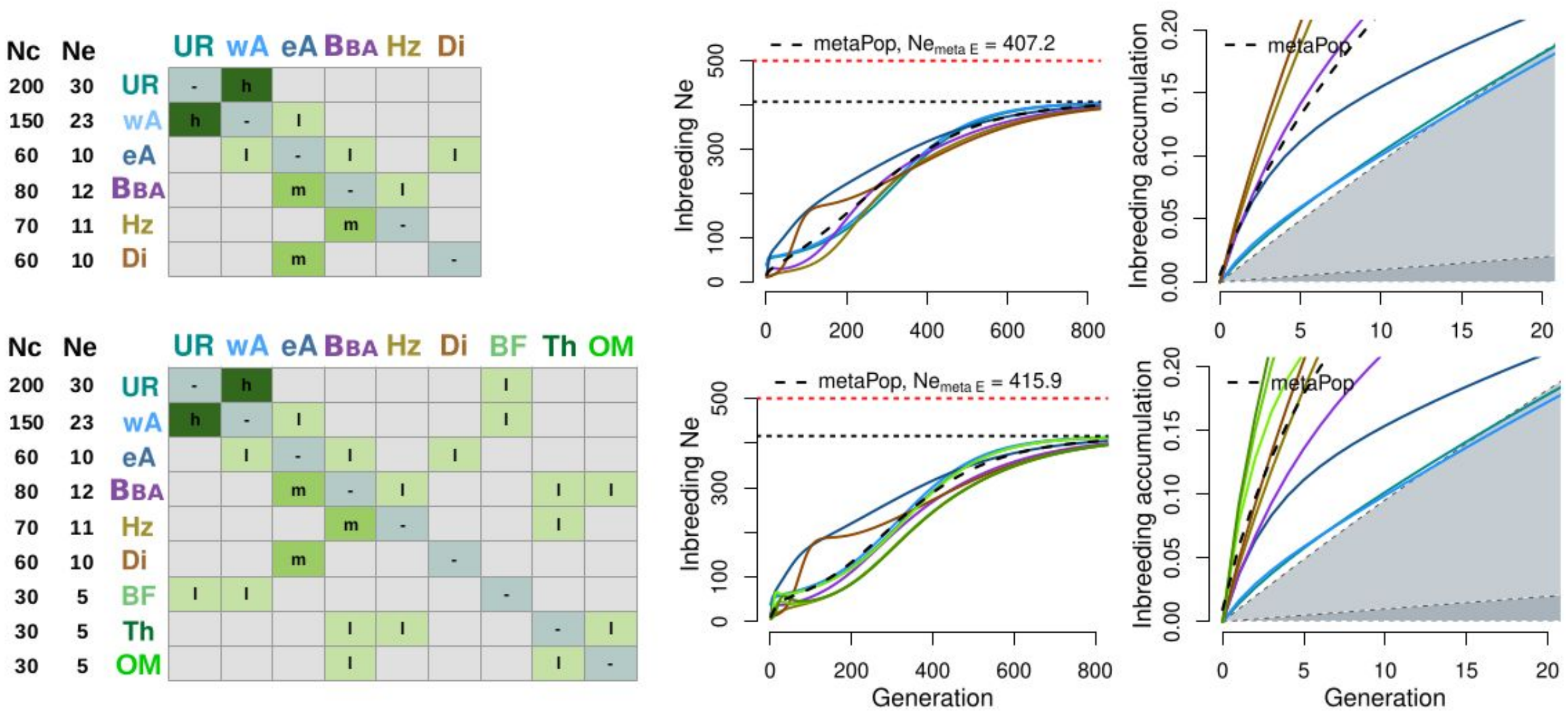

Figure S3 Model outcomes for Sc1 (upper panel) and Sc2 (lower panel), based on a Ne/Nc = 0.15 ratio (see main text). First column: pairwise migration matrix and initial parameters (Nc, Ne), acronyms correspond to main text. Migration rates indicated as low "l", medium "m", and high "h" correspond to Table 1 (x/y depicting from/to direction). Second column: Lines depict effective size (Ne_x) predictions over time, for demes (solid), and the metapopulation (dashed). Horizontal lines depict the Ne_{500} long-term goal (red dots), and the equilibrium effective size $Ne_{meta}E$ of the respective scenario (black dots). Third column: Lines depict inbreeding accumulation over 20 generations in demes (solid), and the metapopulation (dashed); envelopes mark the trajectory of a population of Ne_{50} (light grey) and of Ne_{500} (dark grey) indicating the short- and long-term conservation goal. Colors and acronyms correspond to Fig. 1.

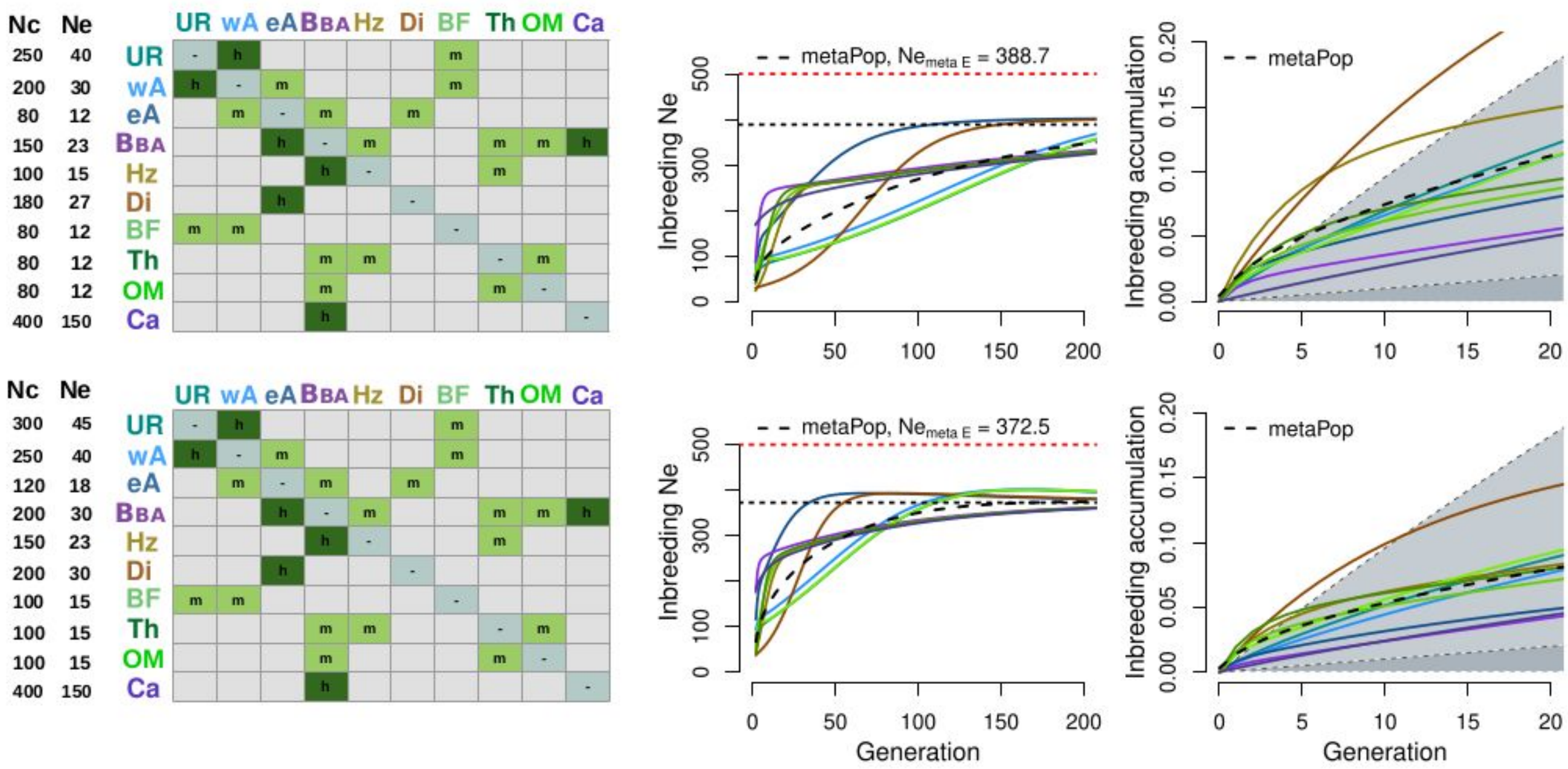

Figure S4 Model outcomes for Sc3 with moderate increase in gene flow (upper panel), and strong increase in gene flow (lower panel), based on a Ne/Nc = 0.15 ratio (see main text). First column: pairwise migration matrix with migration rates indicated as low “l”, medium “m”, and high “h” correspond to Table 1 (x/y depicting from/to direction). Second column: effective size (Ne_x) predictions over time. Third column: inbreeding accumulation over time. Details, see caption Fig. 2.
